# Replacing Hydrogenated Fat in Cookies with Oleogels Based on *Butia odorata* Seed Oil and Beeswax

**DOI:** 10.17113/ftb.63.04.25.8822

**Published:** 2025-12-26

**Authors:** Cristina Jansen-Alves, Elder Pacheco da Cruz, Rosinei Silva Santos, Camila de Oliveira Pacheco, Carem Perleberg, Helen Cristina dos Santos Hackbart, Claudio Martin Pereirade de Pereira, Alvaro Renato Guerra Dias, Elessandra da Rosa Zavareze

**Affiliations:** 1 Laboratory of Biopolymers and Nanotechnology in Food (BioNano), Graduate Program in Food Science and Technology, Department of Agroindustrial Science and Technology, Federal University of Pelotas, 96010-900 Pelotas, RS, Brazil; 2 Laboratory Innovaschem, Bioforensics Research Group, Federal University of Pelotas, 96010-900, Pelotas, RS, Brazil

**Keywords:** beeswax, *Butia* seed oil, fatty acid profile, gas chromatography, texture profile

## Abstract

**Research background:**

Hydrogenated fats are widely used to improve texture, flavor, and shelf life in processed foods, but their excessive consumption contributes to cardiovascular diseases. While *Butia* seed oil contains saturated fats, its potential as an alternative structuring lipid in food applications remains unexplored. This study investigates the formulation of oleogels based on *Butia* seed oil and their potential as a replacement for hydrogenated fats in cookies.

**Experimental approach:**

The aim of this study is to develop oleogels based on *Butia* seed oil and beeswax (*w*=1, 3 and 5 %) and use them in cookies as a substitute for hydrogenated fat. The chemical composition, thermal properties and functional groups of *Butia* seed oil and beeswax were analyzed. The lipid stability, oil binding capacity, gel stability, thermal properties and color parameters of the oleogels were characterized. The oleogels were then added to cookies as a substitute for hydrogenated vegetable fat. Mass loss, color, expansion factor, specific volume and texture properties of the cookies were evaluated.

**Results and conclusions:**

*Butia* seed oil had saturated fatty acids (22.87 mg/mL lauric and 22.45 mg/mL caprylic acid) and notably high concentrations of unsaturated fatty acids (oleic acid 33.21 mg/mL and linoleic acid 30.61 mg/mL). Oleogels containing 3 and 5 % beeswax remained stable for 90 days. Increasing the beeswax mass fraction resulted in greater hardness of the oleogels (p<0.05). Specifically, the oleogel with 5 % beeswax had the highest oil binding capacity, reaching 99.9 %. Cookies formulated with oleogel showed lower hardness and mass loss, as well as a higher specific volume than the control cookie (without oleogel). Notably, the use of oleogels did not alter the visual characteristics of the cookies, supporting their potential as a viable fat replacer in oven-baked products.

**Novelty and scientific contribution:**

These results suggest that oleogels containing *Butia* seed oil have the potential to replace hydrogenated vegetable fats in food products. This study shows that oleogels with *Butia* seed oil, particularly with beeswax mass fractions of 3–5 %, can effectively replace hydrogenated vegetable fats in cookie formulations. Unlike traditional structuring fats, these oleogels offer improved lipid profiles while maintaining desirable baking properties.

## INTRODUCTION

*Butia* sp. (Arecaceae) is a genus of palm trees comprising 20 species found in South America, specifically in Brazil, Paraguay, Uruguay and Argentina (*1*). Brazil is the largest producer of these species (*1*). The fruit of *Butia* sp. is widely consumed in its natural form, and used in artisanal cuisine and production of liqueur (*1*). The seeds are often discarded, used for replanting, as natural fertilizer or as food for local fauna (*2*). Studies emphasize the economic importance of fruit pulp, especially in the pharmaceutical and cosmetic industries (*2*). The seed of *Butia odorata*, often considered a by-product of fruit processing, yields oil rich in lipids. Its fatty acid composition is approx. 76 % saturated fatty acids (including caprylic, capric, lauric, myristic, palmitic and stearic acids) and 24 % unsaturated fatty acids (such as oleic and linoleic acids) (*3*). Despite its rich lipid content, the processing of this oil has received limited attention in scientific research.

Fats found in foods generally consist of a combination of polyunsaturated, monounsaturated and saturated fatty acids (*4*). Saturated fats are widely used in various food products, including bakery items, confectionery, sauces, fast foods and others. These fats provide technological and functional properties to foods, such as flavor, crunchiness and extended shelf life. However, excessive consumption of saturated fats is linked to an increased risk of cardiovascular diseases (*4*).

Conversion of liquid vegetable oils to convert into solid fats, such as hydrogenation, interesterification and fractionation, have been used in the food industry. According to Manzoor *et al*. (*5*), the hydrogenation involves introducing hydrogen atoms into unsaturated fats with a *cis* configuration, converting them into more saturated fats. This conversion results in solid or semi-solid fats with improved properties, such as a higher melting point, increased stability, longer shelf life and greater resistance to oxidation (*5*). However, partial hydrogenation leads to the formation of harmful *trans* fats. In 2018, the US Food and Drug Administration (FDA) announced a ban on partially hydrogenated vegetable oil to eliminate the consumption of foods containing *trans* fats (*6*). Eliminating or replacing *trans* and saturated fatty acids in solid fats has been a challenge for the food industry, due to the importance of fats in texture and flavor (*6*). Consequently, there has been an increased exploration of innovative technologies in this field, such as gelation.

The gelation of liquid oil using structurants (also known as oleogelators) to produce oleogel is considered a viable method for replacing saturated fats (*7*). Oleogels are three-dimensional network structures formed through non-covalent interactions, which restrict the movement of liquid oil and form a solid fat (*6*). Oil gelation with structuring agents involves various interactions, such as hydrogen bonds, electrostatic forces and van der Waals forces (*5*). Oils such as canola (*8*, *9*), corn, soybean, sunflower, olive (*10*, *11*), palm (*12*) and walnut (*13*) have been used in oleogel production. These oils can also serve as a base and act as gelling agents in food systems, such as biscuits and other products, replacing other fats.

Cookies are popular and widely consumed products among various consumer groups. These products typically contain high amounts of fat, ranging from 20 to 30 % based on the mass of the flour (*14*), with saturated fat being commonly used. Kim and Oh (*13*) developed oleogels using oil extracted from an edible insect (*Tenebrio molitor*) together with oleogelators (candelilla wax, carnauba wax and beeswax). They applied them as a substitute for solid fat in cookies. Giacomozzi *et al*. (*14*) formulated muffins using sunflower oil monoglyceride oleogel and compared them to muffins made with commercial margarine. Ghorghi *et al*. (*15*) used grape seed oil to produce oleogel with beeswax as the oleogelator for use in chocolate. However, to our knowledge, no studies have produced oleogel using oil from *Butia* seeds or used this oleogel in food.

The aim of this study is to extract and characterize fatty acids from *Butia* seed oil and assess their application in oleogel production. We also evaluated cookies made with oleogel from *Butia* seed oil and beeswax as a substitute for saturated fat and analyzed their texture, color, expansion factor, specific volume and mass loss.

## MATERIALS AND METHODS

### Material

*Butia odorata* was harvested in 2022 in Rio Grande, RS, Brazil (S 32°10.097' W052°24.595'). The beeswax used to prepare the oleogels was purchased from a local market in the city of Pelotas, RS, Brazil. The reagents used were of analytical grade.

Oil was extracted in a Soxhlet extractor using 20 g of *Butia odorata* seed and 250 mL of hexane p.a. as a solvent. The solvent was evaporated on a rotary evaporator (Büchi®, Rotavapor® RII; Merck, Darmstadt, Germany). The oil extraction yield was calculated gravimetrically based on the initial mass of seeds.

### Fatty acid profile of Butia seed oil

The fatty acid profile was determined using the gas chromatography (GC) method. The equipment is coupled with a GC flame ionization detector (FID) (Clarus 500; PerkinElmer®, Shelton, WA, USA) equipped with a capillary column (30 m×0.25 mm, 0.25 mm Elite-1; PerkinElmer, Waltham, MA, USA). An aliquot of 0.1 mL of *Butia* seed oil was added to 2 mL of hexane and 2 mL of potassium hydroxide. The sample was in an ultrasonic bath for 5 min and then centrifuged for 2 min at 4000×*g* (K14-4000; Kasvi, Paraná, Brazil). The supernatant was collected for analysis (*16*). The operating conditions were as follows: the carrier gas was nitrogen with a constant flow rate of 1.5 mL/min. The column was heated at 120 °C for 1 min, then at a heating rate of 15 °C/min to 160 °C for 3 min, and finally at a heating rate of 3 °C/min to 230 °C and held for 10 min.

Fatty acids were quantified with fatty acid methyl ester (FAME) and a six-point standard curve using mixture C4-C24 (Supelco, Bellefonte, PA, USA) and compounds were identified by comparing elution patterns and retention times with the reference mixture.

### Polar and nonpolar metabolomics analysis of beeswax

For the extraction of polar metabolomics, 0.1 mg of beeswax was placed in a 2-mL bottle, then 1 mL of hexane was added and homogenized using ultrasound for 5 min, and then centrifuged (K14-4000; Kasvi) at 2000×*g* for 5 min. An aliquot of 0.1 mL was then placed in a vial and derivatizated by adding 0.1 mL of N-methyl-N-(trimethylsilyl) trifluoroacetamide. The vial was then placed in a bath at 60 °C for 40 min. Analytes were quantified and identified using a GC system (QP2010 UltraPlus; Shimadzu Corporation, Kyoto, Japan) equipped with a mass detector (MS) (Shimadzu Corporation) and Rxi-1MS capillary column (30 m×0.32 mm×0.25 μm; Restek, Bellefonte, PA, USA). The ramp temperature was maintained at 70 °C for 2 min, increased to 180 °C at 2.5 °C/min, then to 230 °C at 10 °C/min and maintained under isothermal conditions for 2 min. MS was operated in scan mode (mass range *m*/*z*=35–450).

For the extraction of nonpolar metabolomes, 0.1 mg of beeswax was placed in a 2-mL bottle, then 1 mL of hexane was added and homogenized using ultrasound for 5 min, and finally, centrifuged (K14-4000; Kasvi) at 2000×*g* for 5 min. Analytes were quantified and identified using a GC system equipped with a mass detector and an Rxi-1MS capillary column (30 m×0.32 mm×0.25 μm; Restek). The ramp temperature was maintained at 60 °C for 1 min, increased to 180 °C at 5 °C/min, then to 280 °C at 40 °C/min and maintained under isothermal conditions for 15 min (*17*).

### Determination of volatile compounds of Butia seed oil and beeswax

*Butia* seed oil and beeswax were prepared using the headspace solid-phase microextraction method with divinylbenzene/carboxen/polydimethylsiloxane (DVB/CAR/PDMS: 50 μm DVB and 30 μm CAR/PDMS layer) fiber (Supelco, Sigma-Aldrich, Merck, Bellefonte, PA, USA) preconditioned following the manufacturer's protocol. For the extraction of volatile organic compounds (VOCs), 0.1 mL of sample was placed in a 20-mL bottle, then 1 g of sodium chloride and 10 μL of standard benzophenone solution (2 μg) were added. The sealed flasks containing the extract were submerged in a water bath at 40 °C for 15 min, then the fiber was exposed to the headspace for 15 min under constant agitation. VOCs were quantified and identified by a GC system (QP2010 UltraPlus) equipped with a mass detector (Shimadzu), an Rxi-1MS capillary column (30 m×0.32 mm×0.25 μm; Restek) and LabSolution (GCMS solution v. 4.11 SU2; Shimadzu). MS was operated in full scan mode (*m*/*z*=30–450). GC-MS data were analyzed and VOCs were identified by comparing similarity indices and mass spectrum with the NIST11 system database (*18*). Retention index, the retention index calculated from a homologous series of C8-C40 hydrocarbons, and the quantitative analysis was determined by internal standardization with benzophenone (*17*).

### Preparation of the oleogels

The oleogels were prepared according to Lim *et al*. (*19*), with some modifications. Beeswax was added to the *Butia* seed oil at mass fractions of 1, 3 and 5 % and *Butia* seed oil was used as a control. Initially, the *Butia* seed oil was heated in a thermostatic bath to (90±2) °C to ensure complete melting (Fisatom, São Paulo, Brazil) with stirring using a digital mechanical stirrer at 1107×*g* (RW20; IKA, Hamburg, Germany). After reaching the desired temperature, the beeswax was added and stirred for 2 min to dissolve completely and then the samples were placed on an acrylic plate of 32.8 mm (Kasvi). After that, the oil-wax constituents formed an oleogel. Finally, the oleogels were cooled to room temperature ((22±2) °C) and the plates were turned off until subsequent analyses.

### Oil-binding capacity, visual appearance and stability of the oleogels during storage

The oil-binding capacity (OBC) of the oleogels was determined according to the method of Zheng *et al*. (*20*), with some modifications. About 1.5 g of sample was centrifuged (5430 R; Eppendorf Hamburg, Germany) at 7870×*g* for 20 min. The overrun *Butia* seed oil oleogel was then drained onto a soft paper towel for 2 min and the tube mass was weighed together with the solid oil. The empty tube was weighed previously. The values were calculated according to the following equation:


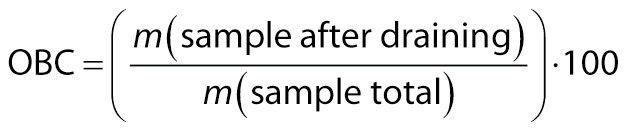
 /1/

**Fig. S1:**
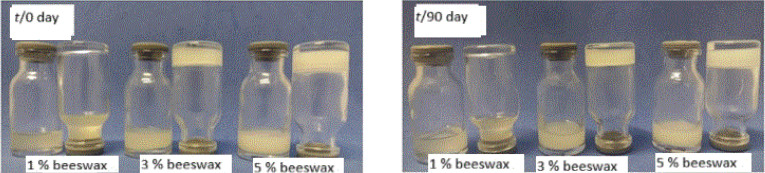
Visual analysis of 90-day storage stability of oleogel *Butia* seed oil with *w*(beeswax)=1, 3 and 5 %

For the stability of the gel during storage, the oleogels based on *Butia* seed oil and beeswax (~4 mL) were put in clear glass vials and placed upright (Fig. S1). Then, the oleogels were stored at 5 °C for 24 h. After that, the oleogels were maintained in a temperature-controlled room with an air conditioner at (22±2) °C for 90 days of analysis, including the phase separation and liquid oil exudation on the surface. The classification was based on the scale on which (*i*) a completely liquid system would be defined as oil-like, with high fluidity and phase separation, (*ii*) a weak system would be characterized by high viscosity, slow flow and minor liquid oil exudation, (*iii*) a medium system showed a visual gel property and could easily flow under gravitational force, (*iv*) a firm system had a visual gel property and could flow slowly under gravitational force, and (*v*) totally firm system had a visual gel property and could not flow under external force (*21*).

### Thermal analyses

The thermal properties of *Butia* seed oil, beeswax and oleogel were analyzed by differential scanning calorimetry (DSC) (Q20; TA Instruments, New Castle, DA, USA). The oleogel (~5 mg) was heated in an aluminum pan at a rate of 10 °C/min. The thermal behavior was evaluated over a temperature range of –20–300 °C. Nitrogen gas with a flow of 25 mL/min was used as the vehicle.

### Fourier transform infrared analyses

The samples of *Butia* seed oil, beeswax and oleogels were evaluated by Fourier transform infrared (FTIR) analysis in conjunction with attenuated total reflection (ATR), using a spectrophotometer model SPIRIT (Shimadzu) scanned from 4000 to 400 cm^-1^, resolution of 4 cm^-1^, and 100 scan readings.

### Oxidative and hydrolytic stability of Butia seed oil and oleogels

The oxidative and hydrolytic stability of samples were evaluated by peroxide index and acidity index, according to AOCS method Cd 8-53 (*22*). The *Butia* seed oil or oleogel (5 g) was dissolved in *V*(acetic acid):*V*(chloroform)=3:2. Starch (1 g/100 g) was used as a starch indicator on the dispersion. The oleogel and *Butia* seed oil were titrated with sodium thiosulfate (0.01 mol/L). The peroxide value was expressed as millimoles of oxygen per kilogram of sample. For acidity index analysis, first, 4 g of the sample were weighed and added to 25 mL of *V*(ether):*V*(alcohol)=2:1. Titration was performed with 0.1 mol/L potassium hydroxide solution. The acidity index was expressed as mg of KOH per gram of sample.

### Preparation of the cookies

The cookies were prepared according to Barragán-Martínez *et al*. (*8*), with modifications, using 100 g wheat flour (containing per 100 g: proteins 14 g, moisture 13 g and ash 0.8 %), refined sugar 44 g, hydrogenated vegetable fat Primor (São Paulo, Brazil), 30 g or *Butia* seed oil 30 g or oleogel 30 g, baking powder (2.2 g; containing: corn starch, chemical leavening agents sodium acid pyrophosphate, sodium bicarbonate and monocalcium phosphate), salt 0.9 g and water 18 g. The oleogel chosen for the cookie formulation was the one with the highest OBC, textural and thermal properties (*Butia* seed oil oleogel with 5 % beeswax). Cookies with the addition of pure *Butia* seed oil and cookies with the addition of hydrogenated vegetable fat (control) were also produced. Hydrogenated fats are chemically formed by two saturated fatty acids, stearic acid and palmitic acid (*23*). The dough was prepared in an electric planetary mixer (Kitchen Aid, Troy, OH, USA). The dry ingredients, part of the flour and hydrogenated vegetable fat were mixed for three minutes at low speed, followed by the addition of water and mixing the dough for one minute at low speed and one minute at medium speed. After adding all the flour, the dough was mixed for two minutes at low speed, rolled to a thickness of 5 mm and cut in a 30-mm diameter. The samples were baked at 180 °C for 9 min in an automatic electric oven (VP 80649; Pratica, Pouso Alegre, Minas Gerais, Brazil). After cooling for 2 h, the physical properties of the cookies were analyzed.

### Mass loss, expansion volume and specific volume of the cookies

Mass loss of the cookies was measured by weighing before and after baking (in grams) using the following equation:


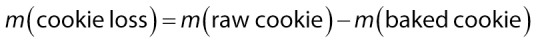
 /2/

The expansion factor was calculated by the ratio between the cookie diameter and thickness values. A caliper was used to measure the thickness and diameter of cookies, using a millimeter scale, 1 h after removal from the oven (*24*). The specific volume of the cookies was calculated as the ratio between the cookie volume (cm^3^), measured by the millet seed displacement method, and the cookie mass after baking. The analysis was done in triplicate.

#### Color parameters of oil and cookies, and texture profile of cookies

The texture profile of the samples was measured using a texturometer (TA.XTplus; StableMicro Systems, Vienna, Austria). The oleogel samples were placed in plastic Petri dishes measuring 90 mm in width and 15 mm height and filled to the brim. A cylindrical probe (20 mm in diameter and 56 mm in length) that was used to compress the oleogel samples at 15 mm/s to a depth of 10 mm (*14*) was positioned at a fixed distance of 20 mm from the base of the equipment. The samples were compressed to 50 % of their initial height by the movement of the probe. The parameters measured were hardness (Newton), springiness, cohesiveness and chewiness. The hardness (N) and fracturability (N) of at least six cookies from each treatment were also evaluated.

The color parameters of the *Butia* seed oil, oleogel and cookies were evaluated by the CIE L*, a* and b* method with a colorimeter (CR-300; Konica Minolta, Osaka, Japan) and illuminator (D65, 10°). The color parameters determined were: L* (dark (0) to light (100)), a* (green (−) to red (+)), and b* (blue (−) to yellow (+)). The chroma (*C*) was the calculated as follows:


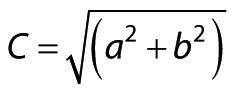
 /3/

For the analysis, five readings were performed on five different positions for each sample.

### Statistical analysis

The values were calculated in triplicate, and the results, expressed as mean value±standard deviation, were analyzed using Statistica v. 7 software (*25*). ANOVA based on factorial design and Tukey’s test (p≤0.05) was used for data analysis.

## RESULTS AND DISCUSSION

### Oil extraction yield and determination of fatty acids

The extraction yield of *Butia* seed oil was (38.4±3.1) % (*m/m*). Hoffmann *et al*. (*1*) reported a 29 to 56 % extraction of *Butia odorata* seed oil. In contrast, Pereira *et al*. (*26*) reported lower values for the extraction of *Butia catarinensis* seed oil, with a yield of 17.2 % (*m*/*m*). As hypothesized, *Butia* seed oil consists predominantly of saturated fatty acids, with a combined concentration of 98.84 mg/mL. Among these, short- and medium-chain fatty acids, including caprylic acid at 22.45 mg/mL and lauric acid at 22.87 mg/mL, are predominant (Table 1). Medium-chain triacylglycerols are less likely to accumulate in the human body than long-chain triacylglycerols due to their rapid absorption into the metabolism, which helps prevent mass gain. These fatty acids are beneficial when requiring a rapid energy source or when there are digestion challenges (*27*).

**Table 1:** Fatty acid profile in *Butia* seed oil

Fatty acid	IUPAC Nomenclature	*γ*/(mg/mL)	*w*/%
C4:0	Butyric acid	10.20	6.43
C8:0	Caprylic acid	22.45	14.15
C10:0	Capric acid	12.56	7.92
C11:0	Undecanoic acid	0.13	0.08
C12:0	Lauric acid	22.87	14.41
C13:0	Tridecanoic acid	0.18	0.11
C14:0	Myristic acid	8.81	5.55
C16:0	Palmitic acid	6.22	3.92
C18:0	Stearic acid	11.25	7.09
C18:1	Oleic acid	33.21	20.93
C18:2	Linoleic acid	30.61	19.29
C20:0	Arachidic acid	0.09	0.06
C24:0	Butyric acid	0.08	0.05
ΣSFA	-	94.84	59.78
ΣMUFA	-	33.21	20.93
ΣPUFA	-	30.61	19.29
ΣTotal	-	158.66	100.00

Σ=sum of saturated fatty acids (SFA), MUFA=monounsaturated fatty acids, PUFA=polyunsaturated fatty acids

Among the unsaturated fatty acids, a high concentration of oleic acid (C18:1) at 33.21 mg/mL and linoleic acid (C18:2) at 30.61 mg/mL was observed. Soybean oil, widely used in food, is primarily composed of linoleic acid (53.2 %) (*23*). In this context, regarding linoleic acid content, *Butia* seed oil exhibits a considerable concentration (30.61 mg/mL). These fatty acids, commonly found in palm fruit oils, have attracted significant interest due to their fatty acid profile, but their application in the oleogel has not yet been studied (*27*). Vieira *et al.* (*28*), however, obtained similar results with higher percentages of lauric acid of 39.17 % in *Butia capitata* oil. Unsaturated fatty acids, such as oleic acid (20.73 %), were also found in *Butia capitata* seed oil (*28*). Pereira *et al*. (*26*) reported that oil extracted from *Butia catarinenses* seeds had the highest content of lauric acid (39.56 %), followed by oleic (11.34 %), capric (10.42 %) and caprylic acid (10.08 %). The composition and quantification of fatty acids depend on various factors, including edaphoclimatic conditions, raw material, cultivation methods, origin of fatty acids, storage conditions and the methods and solvents used (*1*).

The presence of saturated fatty acids provides stability to oxidation, as there are no double bonds in the chain (*28*). Lauric acid, found in significant amounts in *Butia* seed oil, is a short-chain saturated fatty acid known for its antimicrobial properties (*28*). Fats with a fatty acid profile characterized by high percentages of unsaturated and non-*trans* acids remain liquid at room temperature (*29*). Therefore, oleogel based on *Butia* seed oil, which provides a balanced proportion of healthy fatty acids such as oleic acid and linoleic acid (Table 1), is a good solution to replace hydrogenated vegetable fats, thereby reducing *trans* fatty acids in cookie formulations. Moreover, *Butia* seed oil, with a high percentage of linoleic acid (omega 6) and oleic acid (omega 9), is considered significant for food and health, given their roles in cell membranes and brain function, which affect the central nervous system (*1*).

The choice of vegetable oil affects the thermal, textural and rheological properties of the oleogel, thereby influencing its properties and concentration. According to Patel (*30*), oils with different concentrations of saturated and unsaturated fatty acids result in oleogels with different properties. Oils with higher saturated fatty acid content produce oleogels with a firmer texture. Conversely, when the oil used for oleogel preparation has a high content of unsaturated fatty acids, the amount of beeswax must be increased.

### Metabolomic analysis of beeswax

Like many other lipids, beeswax is considered a complex mixture as it consists of a mixture of different class of chemical components, over 300 of them. Each class comprises compounds with different chain lengths, including hydrocarbons, free fatty acids, fatty acid and alcohol esters, diesters and exogenous substances (*31*). Among the chemical compounds in beeswax, fatty acid esters are the main components, formed by combining long-chain fatty acids with long-chain alcohols. Beeswax also contains various hydrocarbons, including medium- and long-chain alkanes, such as triacontane (31.85 %), found in the nonpolar fraction (Table S1), and eicosane, predominantly found in the polar fraction (30.31 %) (Table S2). In addition to esters, beeswax contains free fatty acids, including palmitic acid (24.18 %), linoleic acid (8.26 %) and stearic acid (2.59 %) (Table S2). Long-chain alcohols, such as heptacosanol (1.08 %) and tetracosanol (2.54 %), formed by the reduction of fatty acid esters, are also present in the beeswax. These compounds are part of the nonpolar fraction, with tetratriacontanoic fatty acids comprising 21.92 % and triacontane 31.85 %, making them the major organic compounds.

The use of waxes, particularly beeswax, for structuring edible oils is due to their gel-forming properties at low concentrations and high oil-binding capacity (*32*). Špaldoňová *et al.* (*33*) showed the chromatographic profile of beeswax, which predominantly consists of fatty acid methyl esters with 15-37 carbon atoms, with palmitic acid methyl ester identified as the major compound.

### Volatile organic compounds in Butia seed oil and beeswax

Volatile organic compounds (VOCs) are molecules characterized by high vapor pressure, moderate hydrophilicity and low molecular mass. These compounds play a key role in the sensory properties of foods, affecting aroma and contributing to the acceptance of a product. However, no studies have been conducted on the volatile profile of the oil extracted from *Butia* seed.

The *Butia* seed oil had six volatile organic compounds, four ketones (2-pentanone, 2,3-pentadione, 2-hexanone and sulcatone), one acid (hexanoic acid) and one ester (ethyl hexanoate) (Table 2). The ketones identified in this study are natural compounds commonly found in various plant-derived products. Ethyl hexanoate, with a low aroma threshold, was the main volatile compound in the *Butia* seed oil (23.34 mg/mL). According to Hoffmann *et al.* (*1*), this compound was also found in the pulp of *Butia odorata* and is largely responsible for the aroma associated with this species of *Butia*. Hexanoic acid, a medium-chain volatile fatty acid found in *Butia* pulp, is responsible for off-flavor (unpleasant aroma) (*1*).

**Table 2:** Volatile organic compounds (VOC) identified in *Butia* seed oil

**VOC**	**IS**	**IRL**	**IRL_exp_**	**Reference ion (NIST11)**	**Experimental reference ion**	***γ*/(mg/mL)**
**2-Pentanone**	96	654	-	43.00 (100.00) 86.00 (20.19) 41.00 (11.89)	43.00 (100.00) 86.05 (28.97) 41.00 (11.95)	0.42
**2,3-Pentadione**	91	790	-	43.00 (100.00) 29.00 (61.00) 100.00 (11.89)	43.00 (100.00) 57.00 (58.41) 100.00 (26.66)	1.27
**2-Hexanone**	93	853	938	43.00 (100.00) 58.00 (60.19) 71.00 (13.63)	43.00 (100.00) 58.00 (36.58) 71.05 (13.04)	6.80
**Hexanoic acid**	96	884	1020	74.00 (100.00) 87.00 (31.69) 43.00 (31.29)	74.05 (100.00) 43.00 (43.17) 87.00 (37.95)	3.44
**Sulcatone**	94	938	1071	43.00 (100.00) 41.00 (53.70) 69.00 (35.09)	43.00 (100.00) 41.00 (60.56) 69.05 (37.68)	1.01
**Ethyl hexanoate**	98	984	1092	88.00 (100.00) 99.00 (55.96) 43.00 (44.44)	88.00 (100.00) 99.05 (56.81) 43.00 (54.35)	23.34

IS=index of similarity from Mass Spectral Library of the National Institute of Standards and Technology (NIST) v. 11 (*18*), IRL=index of retention from the NIST11 library (*18*), IRL_exp_=experimental index of retention (based on homologous series *n*-alkane C8-C40). Reference ions (relative intensity, base peak=100 %) from NIST11 library (*18*)

The main volatile compound in beeswax was d-limonene (22.50 %), followed by decanal (9.48 %), α-pinene (9.29 %), nonanal (8.30 %) and β-pinene (8.23 %) (Table 2). In their study on sunflower oleogel with beeswax, Sobolev *et al*. (*34*) identified alcohols such as 1-hexanol, 1-octanol and 1-nonanol, along with the terpene d-limonene. According to Felicioli *et al*. (*35*), the high concentration of decanal aldehyde in beeswax could be associated with antimicrobial activity against *Pseudomonas aeruginosa*. On the other hand, even in small amounts, limonene in beeswax may contribute to antibacterial activity against *Staphylococcus aureus*. Similarly, nonanal may be responsible for the antibacterial properties of beeswax against *Listeria monocytogenes*, even in low concentrations.

### Stability of Butia seed oil and oleogels

The oxidative and hydrolytic stability of the *Butia* seed oil and oleogels were evaluated using the peroxide and acidity indices, respectively. The peroxide content in the oleogel with 5 % beeswax (0.625 mmol/kg) was higher than in the *Butia* seed oil (0.2 mmol/kg) (p<0.05). This result depends on factors such as the number of double bonds in the molecule and the quantity of fatty acids present. The evaluation of lipid oxidation is essential for the determination of the extent of oil oxidation and, consequently, its quality and chemical composition. The extraction of *Butia* seed oil using Soxhlet extraction and the oleogel production at 90 °C are key factors influencing the peroxide and acidity levels in the oil. According to the Codex Alimentarius Commission (*36*), both *Butia* seed oil and oleogels are within the acceptable consumption limit, which is a maximum of 10–15 mmol/kg oil. The initial phase of oil oxidation occurs when fatty acids react with oxygen to form odorless compounds, such as peroxides (*37*).

The acidity index was extremely low for *Butia* seed oil (0.12 mg/g) and higher for the oleogel with 1 % beeswax (0.18 mg/g) (p<0.05). Increasing the beeswax mass fraction to 5 % (0.07 mg/g) in the oleogels effectively reduced the acidity index compared to other oleogels and to the Butia seed oil. Beeswax has emulsifying properties that can help stabilize the oleogel matrix. This stabilization can reduce the exposure of the oil to potential degradation processes, thus lowering the acidity index by minimizing hydrolysis and oxidation reactions that typically generate free fatty acids. The recommended acidity index for refined oils and fats expressed as KOH should not exceed 0.6 mg/g (*36*), indicating that both Butia seed oil and oleogels are suitable for food applications.

### Oil-binding capacity and visual stability of the oleogels

**Fig. 1:**
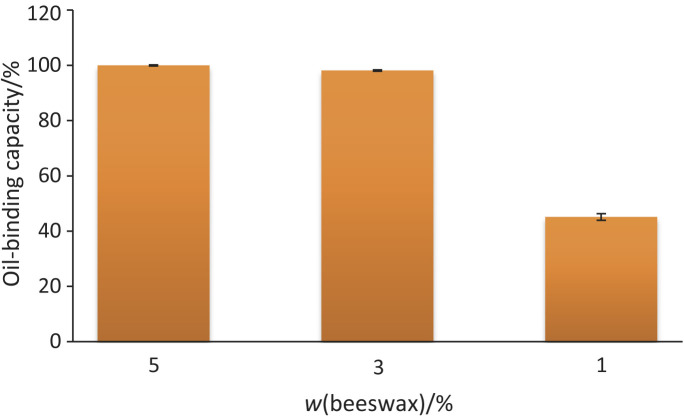
Oil-binding capacity of the oleogel made from *Butia seed* oil and different mass fraction of beeswax

The oil-binding capacity (OBC) values of the oleogel produced with beeswax and *Butia* seed oil are shown in Fig. 1. There was an increase in the OBC with the mass fraction of beeswax, with the highest value observed for the oleogel with 5 % beeswax (99.9 %) and the lowest for the oleogel with 1 % beeswax (45.1 %). Hwang *et al*. (*32*) investigated the effect of the beeswax mass fraction from 0.5 to 15 % on oleogel production. These authors reported that very high mass fractions of wax resulted in a strong flavor and made the emulsion firm, which hindered its incorporation into products and increased the final price of the product with the oleogel. In another study, Zheng *et al*. (*20*) produced an oleogel with oil rich in diacylglycerol. They used ethylcellulose as a gelling agent and added γ-oryzanol as an antioxidant to obtain the results for OBC above 98.5 %. However, increasing the amount of ethylcellulose in the oleogel did not result in a significant increase in the OBC.

Other important factors include the source of vegetable oil, the oleogelator used, and their concentrations, all of which can affect the thermal, textural and rheological properties of the oleogel. Patel (*30*) analyzed five different oils with different concentrations of saturated and unsaturated fatty acids, which gave different results. This author concluded that oils with higher saturated fatty acid concentration form oleogels with a firmer texture. *Butia* seed oil has a higher concentration of saturated fatty acids (98.84 mg/mL) than unsaturated fatty acids (63.82 mg/mL). A higher percentage of saturated fatty acids leads to the formation of a stronger oleogel (*10*).

The visual analysis on the first day showed that the oleogel with 1 % beeswax completely disintegrated, with oil flowing through the tube wall (resulting in 100 % oil release) (Fig. S1). From the first day to the 90th day, the oleogels stabilized with 3 and 5 % beeswax showed no oil loss, remaining stable throughout storage. These results show that the beeswax as a stabilizer in mass fractions above 3 % is effective in absorbing and retaining *Butia* seed oil during storage at room temperature ((20±3) °C). Thus, oleogels produced with 3 and 5 % beeswax had a stable structure for at least 90 days.

### Thermal properties and FTIR analysis

**Fig. 2:**
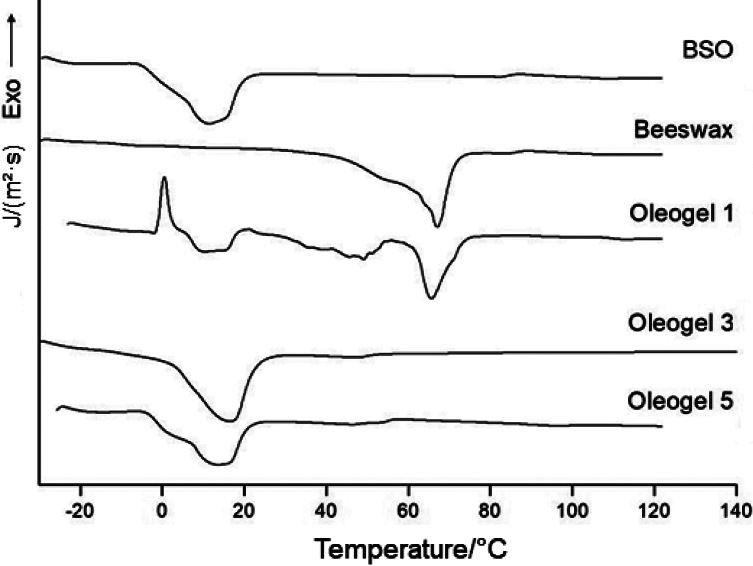
Thermograms of *Butia seed* oil (BSO), beeswax, and oleogel containing *w*(beeswax)=1, 3 and 5 %

The differential scanning calorimetry (DSC) curves of *Butia* seed oil, beeswax and oleogel in the temperature range of -20 to 150 °C are shown in Fig. 2. All thermograms show only one endothermic peak. For *Butia* seed oil, the endothermic peak occurred between 12 and 16.5 °C, with an enthalpy variation of 4.2 J/g. The oleogel showed an endothermic peak starting at 16 to 20 °C, and peaks wider than that of beeswax, with an enthalpy variation of 4.56 J/g. For beeswax, the endothermic peak occurred at around 65.5 °C (melting), with an enthalpy variation of 5.59 J/g^.^

The oleogel had a lower melting point and broader peak than beeswax (Fig. 2). This suggests that *Butia* seed oil contains a variety of compounds, some of which have lower melting points, such as butyric, caprylic and capric acids (Table 1). According to Li *et al*. (*38*), oleogelators with low molecular mass, such as monoacylglycerol, sodium stearoyl lactylate, rice bran wax and beeswax, have melting points at around 60 °C. Waxes are considered among the most efficient structuring agents for the production of oleogels, as they crystallize at low mass fractions added, forming a crystalline network with a high oil retention capacity (*32*, *39*). Hwang *et al*. (*32*) observed a similar change in the melting peak of an organogel containing natural waxes (sunflower wax, rice bran wax, beeswax and candelilla wax). They attributed this phenomenon to the dilution of the wax upon the addition to the oil, which alters the melting temperature of the oleogel. This results in a decrease in temperature and enhances the band signal.

The thermal stability of oleogels is influenced by the stability of the materials used for their stabilization, with enhanced thermal stability typically observed when polymers are combined. Previous studies have documented that oleogels stabilized with certain lipophilic gelling agents, such as fatty acids and waxes, usually have melting temperatures between 50 and 75 °C. Additionally, the melting temperature varies depending on the specific oleogelator used (*39*). However, in this study, the endothermic peaks of both *Butia* seed oil and the oleogel occurred at similar temperatures (16.5 and 20 °C, respectively). This suggests that the crystallization caused by the addition of 5 % beeswax was not significant enough to substantially enhance the thermal stability of the oleogel.

**Fig. 3:**
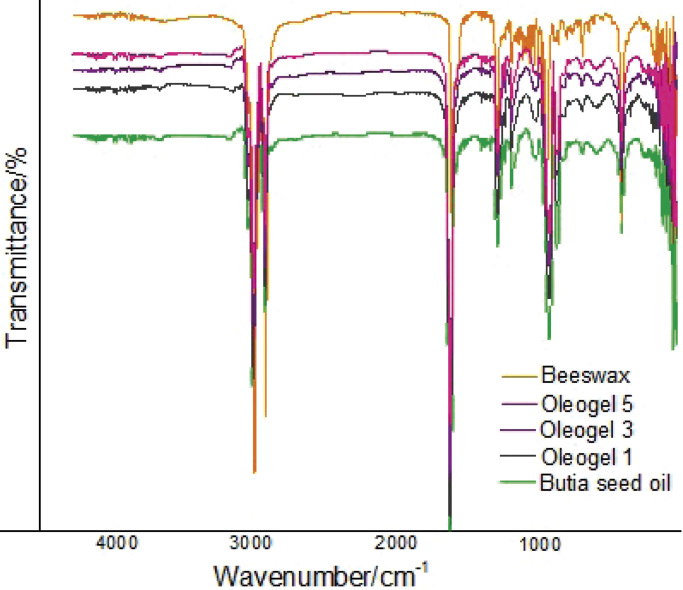
FTIR spectra for beeswax, oleogels containing *w*(beeswax)=5, 3 and 1 % and *Butia* seed oil

Fig. 3 shows the FTIR spectra of *Butia* seed oil and beeswax oleogels at mass fractions of 1, 3 and 5 %. When analyzing the pure compounds (beeswax and *Butia* seed oil), characteristic bands specific to each compound were observed. Bands between 2990 and 2800 cm^-1^, at 2885–2780 cm^-1^ and at 1750 cm^-1^ for *Butia* seed oil, oleogels and beeswax are related to free fatty acids and the methyl group (-CH_3_), methylene group (-CH_2_) and ester group of fatty acids, respectively (*8*). The bands around 1150 cm^-1^ correspond to the stretching of bonds of aliphatic esters and bending vibrations of CH_2_ (*11*); for the spectra of the oleogels with different mass fractions of beeswax, not all characteristic bands of beeswax were identified. This may be due to the low mass fractions of beeswax used.

### Color parameters of the Butia seed oil and oleogels

The color parameters of *Butia* seed oil and oleogels based on *Butia* seed oil containing different mass fractions of beeswax are shown in Table 3. Lightness was higher for *Butia* seed oil (72.46) and decreased with the addition of beeswax, resulting in values of 53.9, 42.7 and 44.1 for oleogels with 5, 3 and 1 % beeswax, respectively. The a* (red/green) coordinate showed negative values for *Butia* seed oil and all oleogels, reflected in greenish tones. Conversely, the b* (yellow/blue) coordinate value increased as the beeswax mass fraction increased. No statistical difference was observed between the *Butia* seed oil and the oleogel with 1 % beeswax regarding the b* coordinate (p>0.05), nor between the oleogels with 3 and 5 % beeswax, indicating that the yellow color of the oleogels is primarily due to the *Butia* seed oil and not the beeswax. *Butia* seed oil contains carotenoids, antioxidant compounds from secondary metabolism, which impart a yellow-orange color (*1*). The chroma value, which measures color purity, was higher for the oleogel with 1 % beeswax and for *Butia* seed oil, with no significant difference between them (p>0.05). However, increasing the beeswax mass fraction (to 3 and 5 %) in the oleogels resulted in a reduction in the chroma value.

**Table 3:** Color parameters of the *Butia* seed oil and oleogels with different mass fractions of beeswax, and texture profile of the oleogels

		Oleogel	
Parameter	Butia seed oil	*w*(beeswax)/%	
		3	5
L*	(72.5±2.2)^a^	(42.7±1.3)^b^	(44.1±1.0)^b^
a*	(-2.9±0.4)^a^	(-0.9±0.2)^c^	(-1.4±0.2)^b^
b*	(13.2±1.4)^a^	(6.9±0.1)^b^	(5.2±0.9)^b^
C*	(13.6±1.4)^a^	(6.9±0.1)^b^	(5.4±0.8)^c^
Hardness/N	_	(5.8±0.4)^b^	(22.3±1.1)^a^
Springiness	_	(0.99±0.00)^a^	(1.00±0.00)^a^
Cohesiveness	_	(0.38±0.02)^a^	(0.36±0.02)^a^
Chewiness	_	(22.6±0.9)^b^	(81.7±0.8)^a^

Results are expressed as mean value±standard deviation (*N*=3). Different letters in the same row are significantly different for color parameters evaluated by Tukey's test and (p<0.05). Different letters in the same row are significantly different for texture profile evaluated by *t*-test (p<0.05)

### Texture profile analysis of the oleogels

The texture profile analysis of the oleogels is shown in Table 3. Hardness, defined as the maximum force during the first compression, was the highest in the oleogel with the highest mass fraction of beeswax compared to the other samples (p<0.05). The oleogel with the lowest beeswax mass fraction (1 %) showed greater springiness than the other formulations (p<0.05). Overall, all oleogels produced in this study had low springiness values, indicating a limited ability to return to their original form after deformation, a property inherent to oleogels. Fats are classified by their plasticity, where lower elasticity is associated with greater plasticity (*32*). The springiness and cohesiveness values of the oleogels containing 3 and 5 % beeswax were quite similar, both lower than that of the oleogel with 1 % beeswax, which had the highest values. In terms of hardness and chewiness, the oleogel with 5 % beeswax showed the highest values, which is in agreement with the high OBC results observed (Fig. 1).

Beeswax has a diverse chemical composition, containing esters, hydrocarbons (eicosane), free fatty acids (palmitic, linoleic, oleic and stearic), and free fatty alcohols (tetracosanol). These compounds, particularly the esters with long chains, crystallize and form networks that trap the liquid vegetable oils. This crystallization changes the physical properties of the oil, affecting the elasticity and hardness of the oleogel (*32*). The texture profile of oleogels is crucial for their optimal industrial application.

### Application of the Butia seed oil and oleogels in the cookies

**Fig. S2:**
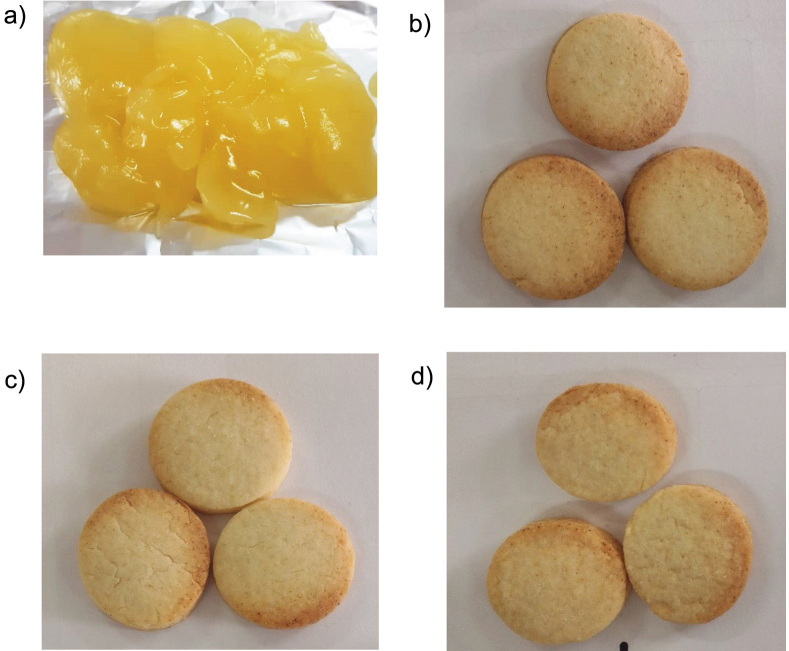
Oleogel containing *w*(beeswax)=5 % and *Butia* seed oil (a); cookies made with: b) hydrogenated vegetable fat, c) oleogel, and d) *Butia* seed oil

The evaluation of texture, color, mass loss, expansion factor and specific volume of the cookies made with hydrogenated vegetable fat (control), *Butia* seed oil and oleogel containing *Butia* seed oil and 5 % beeswax is shown in Table 4 and Fig. S2. Oleogel with 5 % beeswax was selected for cookie production due to its gel stability and high oil-binding capacity, ensuring the maintenance of the desired cookie texture.

**Table 4:** Texture, color and physical parameters of cookies produced with hydrogenated vegetable fat (control), *Butia* seed oil (BSO), and oleogel

	Cookie		
Parameter	Hydrogenated vegetable fat (control)	*Butia* seed oil	Oleogel (BSO and *w*(beeswax)=5 %)
	Texture profile		
Hardness/N	(1103±60)^a^	(897±68)^b^	(723±49)^c^
Fracturability/N	(0.6±0.1)^a^	(0.6±0.1)^a^	(0.70±0.04)^a^
L*	(79.6±1.6)^a^	(78.6±0.8)^a^	(78.9±1.2)^a^
a*	(0.5±0.4)^a^	(0.4±0.3)^a^	(0.3±0.4)^a^
b*	(22.3±1.9)^a^	(22.2±1.1)^a^	(22.6±1.6)^a^
C*	(22.3±1.9)^a^	(22.4±1.1)^a^	(22.6±1.6)^a^
*m*(loss)/%	(14.1±0.5)^a^	(12.2±0.8)^b^	(10.8±0.7)^c^
*v*/(g/cm^3^)	(0.81±0.02)^b^	(0.92±0.02)^b^	(1.3±0.2)^a^
Expansion factor	(4.8±0.2)^a^	(4.0±0.2)^c^	(4.5±0.2)^b^

Different letters in the same row are significantly different for each parameter evaluated by Tukey's test (p<0.05)

The cookies made with the oleogel showed the lowest hardness, followed by the cookies with *Butia* seed oil, and those with hydrogenated vegetable fat (control). Hardness refers to the ability to resist deformation under applied force. The fracturability (or brittleness) of a cookie is the distance it deforms until it breaks under stress (*34*, *40*). Other authors have obtained lower values for fracturability of cookies containing oil gels modified with olive oil, soybean oil, linseed oil, and wax, than in this study (*40*).

In this study, cookies made with *Butia* seed oil had lower hardness than those made with hydrogenated vegetable fat. The reduced hardness of the cookie produced with oleogel and pure *Butia* seed oil may be attributed to the chemical composition of the *Butia* seed oil and the beeswax present in the oleogel formulation. The primary components of beeswax are fatty acid esters, which act as emulsifiers, along with monoglycerides and fatty acids (stearic acid and oleic acid) (*32*). The presence of these emulsifiers can improve the incorporation of air into the dough and thus reduce its hardness.

No significant difference was observed in the fracturability of the cookies (p>0.05). Fracturability is related to crispness and is a key factor for cookies. Fat affects the softness of the cookies by lubricating the dough, while sugar impacts the characteristics of fracturing or breaking. The thermal stability of the materials and their melting temperature used in cookie production also influence the texture properties (*22*).

The fracturability (or brittleness) of the biscuit is defined as the deformation (in millimeters or centimeters) that the sample undergoes until it breaks under particular conditions (*40*). Despite the relatively small thickness of the biscuit, a force 10 to 12 times greater is required for it to fracture. The applied force influences the positioning of the probe, causing it to travel a greater distance before rupture occurs. It is important to note that the texture analysis was conducted 1 h after baking. Until that time, the biscuits had already cooled and had a dry structure with no residual moisture. This condition contributed to the increased hardness and the reduced fracturability (*40*).

Hydrogenation, or the use of saturated fats, is necessary to structure liquid oil into semi-solid plastic pastes for use in spreads, margarine and shortenings (*40*). However, hydrogenated fats are composed primarily of saturated fatty acids, such as palmitic and stearic acid (*23*). Bakery products have been the focus of studies that aim to replace hydrogenated fats with structured oils, due to the global rise in overweight and obesity associated with the high consumption of unhealthy, energy-dense diets, including processed foods (*8*). Zhao *et al*. (*41*) used oleogels in an attempt to replace commercial fat in biscuits. He concluded that biscuits with corn oil oleogel had properties comparable to the control (visual appearance, hardness and firmness), resulting in healthier biscuits with a high content of unsaturated fats.

There was no significant difference in the color parameters between the cookies made with oleogel, *Butia* seed oil, and hydrogenated vegetable fat (p>0.05). Visual analysis showed that all cookies had a spherical shape with some small grooves on the surface and the color (Table 4 and Fig. S2) was predominantly yellow (Fig. S1 and Fig. S2). The cookies made with oleogel had the lowest mass loss during baking, followed by the cookies with *Butia* seed oil (p<0.05), while the cookies with hydrogenated vegetable fat had the highest mass loss (p<0.05). These results indicate that using oleogel increased mass yield during baking. Goldstein and Seetharaman (*42*) reported a relationship between cookie height and moisture content in the cookies made with a monoglyceride emulsion, where greater height was associated with increased moisture. Moisture loss affects cookie volume and expansion capacity, which may explain the lower expansion factor observed in the cookie made with *Butia* seed oil (4.01, p<0.05) (Table 4), as no gel was formed to retain moisture during baking.

The cookies made with oleogel had the highest specific volume, while the cookies with *Butia* seed oil and the control did not differ from each other (p>0.05). There was an inverse relationship between specific volume and hardness: the cookies with oleogel had the highest specific volume (1.3 g/cm^3^) and the lowest hardness. In contrast, the cookies made with hydrogenated vegetable fat had the lowest specific volume (0.81 g/cm^3^) and the highest hardness compared to those made with *Butia* seed oil and oleogel. These results may be related to the higher mass loss observed in these cookies, 14.1 % (p<0.05) (Table 4). Specific measurements of volume, thickness and width in cookies are influenced by several factors, including ingredient quality, texture (softness or hardness), types and quantities of ingredients, and processing conditions.

Regarding the expansion factor, there was a significant difference between all cookies, with the highest expansion factor observed for the control cookies. Factors such as cookie diameter, thickness and expansion factor are commonly used to assess product quality. According to Jacob and Leelavathi (*40*), the spreading of the cookie dough is related to the viscosity of the dough since a dough with low viscosity takes longer to stop spreading in the mold and harden, leading to larger sizes. However, cookies with very high or very low expansion factors cause problems in the processing of industrialized foods, resulting in products with low or high mass.

Oleogelation is a promising method, as it does not alter the unsaturated fatty acid profile of liquid oils. The replacement of 100 % hydrogenated fat with *Butia* seed oil oleogel containing beeswax aims to reduce the content of saturated and hydrogenated fats, as well as to improve the lipid profile of cookies, since *Butia* seed oil is rich in polyunsaturated fatty acids such as linoleic and oleic acids (Table 1). Furthermore, the oleogel production process does not use chemical reagents to transform liquid oils into thermo-reversible and three-dimensional gel networks with solid-like properties.

## CONCLUSIONS

*Butia* seed oil, considered an unexplored source, contains saturated fatty acids such as lauric acid and caprylic acid, as well as unsaturated acids including oleic and linoleic acids. The main volatile component of *Butia* seed oil is d-limonene. Different mass fractions of beeswax added to the oleogel altered the interactions between *Butia* seed oil and beeswax, thereby changing the properties of the oleogel. Oleogels containing 3 and 5 % beeswax remained stable over 90 days of storage. The increase in the mass fraction of beeswax increased the hardness of the oleogels, while the production process did not adversely affect the quality of *Butia* seed oil. Oleogel formulated with 5 % beeswax and *Butia* seed oil was used in cookies as a replacement for hydrogenated vegetable fat. Cookies containing oleogel showed reduced hardness and mass loss, demonstrating potential as a substitute for saturated and trans fats in food products.

However, challenges remain regarding scale-up production, stability under varying industrial storage conditions, and regulatory approval for new lipid structures. Future studies should also explore its functional performance in diverse food systems and processing environments to validate its technological viability and consumer acceptance. A formulation with biological activity, such as antimicrobial and antifungal properties, would be ideal for applying these oils in bakery products.

## Appendix

**Table S1:** Polar fraction of the compounds identified in the beeswax

Polar fraction	IS	IRL	IRL_exp_	Reference ion (NIST11)	Reference ion exp	*w*/%
13-Octadecenoic acid, (E)-, TMS	93	2194	2227	73.00 (100), 75.00 (93.89), 55.00 (81.38)	73.05 (100), 75.00 (86.20), 55.05 (44.33)	1.18
1-Nonadecene	90	1900	1938	97.00 (100), 83.00 (92.94), 43.00 (91.76)	97.10 (100), 83.10 (86.60), 43.05 (52.80)	0.42
9-Hexadecenoic acid, (Z)-, TMS	94	1995	2017	117.00 (100), 73.00 (98.60), 75.00 (97.70)	73.05 (100), 17.05 (96.28), 75.00 (89.53)	2.08
Arachidic acid, TMS	90	2385	2416	73.00 (100), 117.00 (76.58), 75.00 (67.22)	117.05 (100), 73.05 (77.09), 75.00 (59.93)	0.72
Docosane	95	2208	2200	57.00 (100), 71.00 (71.33), 43.00 (63.42)	57.10 (100), 71.10 (91.67), 43.05 (58.39)	8.97
Eicosane	95	2009	2010	57.00 (100), 71.00 (77.28), 43.00 (63.07)	57.10 (100), 71.10 (91.67), 43.05 (58.39)	30.31
Elaidic acid, (E)-, TMS	94	2194	2215	73.00 (100), 117.00 (95.00), 75.00 (94.60)	117.05 (100), 73.05 (94.12), 75.00 (78.03)	14.83
Heneicosane	96	2109	2100	57.00 (100), 71.00 (70.27), 85.00 (54.95)	57.10 (100), 71.10 (77.11), 85.10 (55.77)	1.34
Heptacosanol	94	2948	3016	57.00 (100), 97.00 (97.00), 83.00 (95.20)	97.10 (100), 83.10 (95.93), 57.10 (79.03)	1.08
Linoleic acid, TMS	94	2202	2208	75.00 (100), 73.00 (97.90), 67.00 (65.07)	73.05 (100), 75.00 (82.12), 67.05 (60.60)	8.26
Methyl stearate	91	2077	2102	74.00 (100), 87.00 (74.48), 43.00 (33.73)	74.00 (100), 87.05 (60.32), 43.05 (26.18)	1.50
Palmitic acid, TMS	93	1987	2036	117.00 (100), 73.00 (86.09), 313.00 (68.97)	117.05 (100), 73.05 (81.64), 313.20 (59.32)	24.18
Stearic acid, TMS	92	2186	2234	117.00 (100), 73.00 (77.08), 341.00 (67.17)	117.05 (100), 73.05 (77.72), 341.25 (55.38)	2.59
Tetracosanol	94	2650	2700	57.00 (100), 83.00 (95.40), 55.00 (94.70)	83.10 (100), 57.05 (78.64), 55.05 (63.11)	2.54

IS=index of similarity from NIST11 (National Institute of Standards and Technology) library, IRL=index of retention from NIST11 library, IRL_exp_=experimental index of retention (based on homologous series *n*-alkane C8-C21). Reference ions (relative intensity, base peak=100 %) from NIST11 library and reference ions experimental. TMS=trimethylsilyl ester

**Table S2:** Nonpolar fraction of the compounds identified in the beeswax

Non-polar fraction	IS	IRL	IRLexp	Reference ion (NIST11)	Reference ion exp	*w*/%
9-Hexacosene	92	2614	2566	43.00 (100), 97.00 (97.06), 57.00 (96.53)	97.10 (100), 43.05 (86.25), 57.05 (69.51)	9.09
E,E,Z-1,3,12-Nonadecatriene-5,14-diol	90	2241	2200	55.00 (100), 95.00 (85.17), 81.00 (79.74)	81.05 (100), 55.05 (84.98), 95.10 (75.51)	1.13
Eicosane	96	2009	2000	57.00 (100), 71.00 (77.28), 43.00 (63.07)	57.05 (100), 71.10 (94.88), 43.05 (61.42)	1.80
Hexatriacontane	93	3600	3400	57.00 (100), 71.00 (74.40), 43.00 (54.90)	57.05 (100), 71.10 (99.78), 43.05 (56.6)	6.66
Methyl elaidate	95	2085	2084	55.00 (100), 69.00 (95.50), 74.00 (90.20)	55.05 (100), 69.05 (86.55), 74.05 (72.32)	0.42
Methyl palmitate	93	1878	1920	74.00 (100), 87.00 (72.07), 43.00 (32.53)	74.00 (100), 87.05 (55.29), 43.05 (22.05)	0.88
Methyl stearate	92	2077	2109	74.00 (100), 87.00 (76.48), 298.00 (35.23)	74.05 (100), 87.05 (61.54), 298.25 (26.97)	1.84
Neoheptanol	86	875	868	43.00 (100), 85.00 (51.01), 41.00 (29.00)	43.05 (100), 85.10 (40.14), 41.05 (20.72)	0.22
Nonadecyl trifluoroacetate	94	2110	2100	57.00 (100), 97.00 (97.40), 83.00 (84.49)	97.10 (100), 83.10 (87.39), 57.05 (72.44)	2.51
Olean-12-en-3-ol	90	2886	2970	218 (100), 203 (43.84), 219.00 (18.02)	218.15 (100), 103.10 (54.22), 219.15 (18.02)	0.36
Oleic acid	90	2175	2171	55.00 (100), 69.00 (81.98), 83.00 (77.58)	55.05 (100), 69.05 (94.33), 83.10 (74.97)	1.20
Oleic acid amide	90	2228	2375	59.00 (100), 72.00 (50.30), 55.00 (22.69)	59.00 (100), 72.05 (77.13), 55.05 (25.42)	3.69
Palmitic acid	91	1968	1967	60.00 (100), 73.00 (98.10), 57.00 (84.09)	73.05 (100), 60.00 (66.88), 57.05 (62.71)	2.93
Tetracosane	94	2407	2400	57.00 (100), 71.00 (68.00), 43.00 (56.20)	57.05 (100), 71.10 (91.59), 43.05 (59.00)	12.32
Tetratriacontane	95	3401	3400	57.00 (100), 71.00 (71.20), 43.00 (54.40)	57.05 (100), 71.10 (73.68), 85.10 (63.35)	21.92
Triacontane	95	3003	3000	57.00 (100), 71.00 (72.10), 85.00 (53.50)	57.05 (100), 71.10 (82.81), 85.10 (68.39)	31.85
Trimethylsilyl palmitate	93	1987	2041	117.00 (100), 73.00 (86.09), 313.00 (68.97)	117.05 (100), 73.05 (74.56), 313.20 (60.08)	1.18

IS=index of similarity from NIST11 (National Institute of Standards and Technology) library, IRL=index of retention from NIST11 library, IRL_exp_=experimental index of retention (based on homologous series *n*-alkane C8-C21). Reference ions (relative intensity, base peak=100 %) from NIST11 library and experimental reference ions
